# Rate-Control or Rhythm-Control: Where do we stand?

**Published:** 2005-10-01

**Authors:** L Testa, G Trotta, A Dello Russo, M Casella, G Pelargonio, F Andreotti, F Bellocci

**Affiliations:** Institute of Cardiology, Catholic University, Rome, Italy

**Keywords:** atrial fibrillation, rate control, rhythm control, secondary prevention

## Abstract

Atrial fibrillation is the most common sustained rhythm disturbance and its prevalence is increasing worldwide due to the progressive aging of the population. Current guidelines clearly depict the gold standard management of acute symptomatic atrial fibrillation but the best-long term approach for first or recurrent atrial fibrillation is still debated with regard to quality of life, risk of new hospitalizations, and possible disabling complications, such as thromboembolic stroke, major bleeds and death. Some authors propose that regaining sinus rhythm in all cases, thus re-establishing a physiologic cardiac function not requiring a prolonged antithrombotic therapy, avoids the threat of intracranial or extracranial haemorrhages due to Vitamin K antagonists or aspirin. On the contrary, advocates of a rate control approach with an accurate antithrombotic prophylaxis propose that such a strategy may avoid the risk of cardiovascular and non cardiovascular side effects related to antiarrhythmic drugs. This review aims to explore the state of our knowledge in order to summarize evidences and issues that need to be furthermore clarified.

## Background

Atrial fibrillation (AF) is the most common serious rhythm disturbance worldwide [1] and its incidence progressively increases with the aging of population by two fold with every decade after 55 year of age [2]. It is associated with increased morbidity and mortality [3], and it is an independent risk factor for stroke: AF increases by 5-fold the overall risk of thromboembolic stroke (TES) and by 12-15% in high risk subsets where further risk factors as hypertension, diabetes mellitus, heart failure, or previous transient ischemic attack, are present [4]. A variety of symptoms such as reduce exercise tolerance, palpitations, dizziness, dyspnoea or other signs of heart failure able to heavily affect the quality of life, may be related to AF [5-8]. Current guidelines clearly state the gold standard approach for symptomatic atrial fibrillation with haemodynamic impairment but, on the contrary, whether rate control approach is preferable to rhythm control strategy is still debated.

## An Unresolved Dilemma

Traditionally, a rhythm control approach with electrical or pharmacological cardioversion followed by antiarrhythmic drug prophylaxis was preferred by physician even if strong evidence of its superiority have never been available [9]. Theoretically, restoration and maintenance of sinus rhythm holds some advantages as it re-establishes a physiologic cardiac function, avoids the unfavourable ventricular and atrial remodelling[10,11] due to prolonged tachycardia, and reduce the risk of thromboembolic stroke resuming a normal atrial systole. Current guidelines advocate anticoagulation for 3-4 weeks before and after cardioversion of AF of >48 hours' duration. Alternatively, early cardioversion without anticoagulation may be performed after exclusion of left atrial thrombi by transesophageal echocardiography [1]. Thus, even if evidence of asymptomatic recurrences is growing [12], as of now, prolonged anticoagulation is not yet recommended after restoration of sinus rhythm, due to the known increased risk of haemorrhagic stroke or other major and minor haemorrhages due to Vitamin K antagonists [13,14]. On the other hand, currently available antiarrhythmic drugs have many side effects which in some cases may be life threatening as they are proarrhythmic. Amiodarone is the drugs more frequently used in published trials but also in clinical practice; noteworthy, some reports stated its relation with increased non cardiovascular mortality [15-17]. Advocates of a simple rate control approach associated with an accurate antithrombotic regimen propose that such a strategy would not only avoid the proarrhythmic risk of anti arrhythmic drugs but also ensures a better outcome due to the beneficial effect of warfarin therapy [18]. Rate control approach seems to be equally effective in improving quality of life (QoL) respect to rhythm control approach even if the major predictor of QoL improvement was sinus rhythm [19,20].

## Rate Control Strategy

Generally, a rate control approach is considered effective when associated with relief of symptoms and when mean ventricular response ranges between 60 and 80 beats per minute (bpm) at rest and between 90 and 115 bpm during a common moderate exercise test [1,21]. Beta blockers, verapamil, diltiazem, digoxin but also amiodarone are effective in reducing the heart rate: beta blockers are the drugs of choice in patients with coronary artery disease and, in presence of systolic dysfunction, they may be even more valuable [22]. Verapamil is able to rise the serum digoxin levels so the dosage of the latter must be reduced if administered with verapamil [23]. As sole therapy, digoxin may be suitable for elderly patients [24]. Amiodarone is highly effective in controlling the heart rate but many concerns have been raised about its long-term safety [15-17].

The “ablate and pace” approach consists of the ablation of atrioventricular node followed by implantation of a permanent pacemaker. The ventricular rate is completely controlled by the pace-maker and also a more physiological contraction of the ventricle is restored thus positively affecting the cardiac haemodynamic [25]. As the atria continue to fibrillate, the need of anticoagulant therapy remains unchanged. In patients with heart failure and refractory to other treatment, this approach was found to be associated with better control of palpitations, improvements of dyspnoea and quality of life [26]. No improvement of cardiac performance [26] neither prolonged survival [27] were observe with “ablate and pace” strategy respect to pharmacological therapy. In some cases a modulation instead of ablation of atrioventricular node may be efficacious to improve symptoms [28].

## Rhythm Control Strategy

Current guidelines state which antiarrhythmic drugs have proven efficacy in converting atrial fibrillation into sinus rhythm both for atrial fibrillation with less and more than 7 days of duration [1]. Also which drugs are effective in increasing the success rate of electrical cardioversion are described[1]. All of these antiarrhythmic drugs are burdened by the risk of serious cardiovascular and non cardiovascular side effects, including life threatening ventricular arrhythmias (all), lupus like syndrome (procainamide), heart failure (disopyramide, propafenone, flecainide, sotalol), pulmonary toxicity (amiodarone, sotalol). A recent meta analysis supports the efficacy of amiodarone in converting persistent atrial fibrillation [29] but its known thyroid, hepatic, and pulmonary toxicity should make it a second line agent [30].

Electrical cardioversion has a very high initial success rate, but only about 23% of patients at 1 year and 15% at 2 years remain in sinus rhythm [31][32]. Many studies investigated the clinical and instrumental factors able to predict the risk of relapse; the most common accepted are: AF duration more than 1 year, age > 75 year, heart failure and increased atrial dimension [33].  Currently, for patients with unknown atrial fibrillation duration or more than 48 hours, cardioversion is recommended after a 3 weeks period of anticoagulation and it has to be followed by a month of anticoagulation [1]. A transoesophageal echocardiography-guided approach showed a similar risk of stroke compared to conventional management [34] thus suggesting that the risk of stroke is not completely abolished restoring sinus rhythm, even if transoesphageal examination is negative for intra atrial clot.

Recently, newer strategies were developed, including catheter ablation by means of radiofrequency. Ectopic foci, localized within the pulmonary vein, were found in the majority of atrial fibrillation [35] and the ablation of this triggers, despite a little but predictable risk of iatrogenic morbidity [36], seems to be very promising in light of a chance of “real” cure for atrial fibrillation [37]. Device-based therapy [38] but also surgical treatment with the Cox-Maze procedure have been developed in the last years with some promising results [39].

## Randomized Trials of Rate vs Rhythm

Several trials addressing this issue were published in the last years [40-45] and two others are still ongoing [46,47]. The Pharmacological Intervention in Atrial fibrillation (PIAF) PIAF) [40] enrolled 252 patients with persistent atrial fibrillation duration up to 360 days. In the first arm the strategy was to control heart rate using diltiazem while, in the second, restoration and maintainance of synus rhythm was obtained using amiodarone or electrical cardioversion as first intervention followed by various antiarrhythmic drugs.  Follow up length was 1 year. The primary end point of the study was improvement in symptoms related to atrial fibrillation.

The Strategies of Treatment of Atrial Fibrillation study (STAF) [41] enrolled 200 patients with persistent atrial fibrillation. In the rhythm control arm patients were to be cardioverted by external or internal cardioversion; after restoration of sinus rhythm, prophylaxis was performed with class I antiarrhythmic drugs or sotalol, in the absence of coronary artery disease (CAD) and in the presence of normal left ventricular ejection fraction, while in the presence of impaired ventricular function or CAD, beta-blockers or amiodarone were used.  In the rate control arm beta-blockers, digitalis, calcium antagonists, or atrioventricular node ablation/modification with or without pace maker implantation were used. Follow up was of 19.6 + 8.9 months. Primary end point was the combined rate of death, cardiopulmonary resuscitation, cerebrovascular events and systemic embolism.

The RACE study [42] enrolled 522 patients with persistent atrial fibrillation after an initial attempt of electrical cardioversion. Rate control was achieved with administration of digitalis, calcium antagonists, and beta-blockers alone or in combination. Rhythm control was obtained with electrical cardioversion without previous treatment with antiarrhythmic drugs. Thereafter, sotalol was used for prophylaxis. At the first recurrence of atrial fibrillation, electrical cardioversion was repeated and sotalol replaced by flecainide. In the presence of a recurrence within 6 months another cardioversion was performed and flecainide replaced by amiodarone. Follow up length was 2.3 + 0.6 years. The primary end point was a composite of cardiovascular death, heart failure, thromboembolic complications, bleeding, implantation of a pace-maker, and severe adverse effects of drugs.

The AFFIRM study [43] enrolled 4060 patients with first or recurrent atrial fibrillation at high risk for stroke in a randomized, multicenter comparison. Risk factors for stroke were considered hypertension, diabetes mellitus, congestive heart failure, prior transient ischemic attack, cerebrovascular accident, systemic embolism history, left atrial size of 50mm or more, LVEF less than 40%. Digitalis, calcium antagonists, and beta-blockers alone or in combination were the drugs accepted in the rate control arm: the goal was a heart rate not higher than 80 beats per minute at rest and 110 beats per minute during six minute walk test. In the rhythm control arm the antiarrhythmic drug was chosen by the treating physician: attempts to maintain rhythm control could include cardioversion. The following drugs were acceptable: amiodarone, disopyramide, flecainide, moricizine, procainamide, propafenone, quinidine, sotalol, and their combination according to an imposed protocol. Mean follow up length was 3.5 years. The primary end point was overall mortality but several other clinical end-points were reported.

The HOT CAFÉ [44] enrolled 205 patients with a mean time of atrial fibrillation duration of 273 ± 112.4 days. In the rate control arm beta blockers, calcium antagonists, digoxin alone or in combination were the pharmacological treatment. Patients randomised to rhythm control strategy were all treated with electrical cardioversion and subsequent antiarrythmic drugs. Follow up length was 1.7 ± 0.4 years. Primary end point was a composite of death from any cause, thromboembolic complications (especially disabling stroke), and intracranial or other major haemorrhage.

The Control of Rate versus Rhythm in Rheumatic Atrial Fibrillation trial (CRRAFT) [45] trial differs from the others because it enrolled patients with rheumatic heart disease and chronic atrial fibrillation. Forty-eight patients randomized to rate control received 90 mg sustained release diltiazem twice daily to maintain the resting ventricular rate below 90 beats/min, and less than 130 beats/min with activity. No attempt to restore SR with drug therapy or electroversion was made. Those who entered the rhythm control group were further randomized in a double-blind design to receive either amiodarone or placebo and electrical conversion, wherever required. Follow up length was 1 year. Primary end points were: exercise tolerance assessed by Bruce protocol treadmill exercise, NYHA class, QOL score, thromboembolic and bleeding complications, hospitalization rates, and deaths. While the CRRAFT trial suggested that maintenance of sinus rhythm appeared to be superior to ventricular rate control in patients with rheumatic atrial fibrillation in terms of an effect on mortality and morbidity, none of the single published trials on non-rheumatic atrial fibrillation shows statistically significant difference between the two strategies, only suggesting a possible superiority of rate control approach in terms of a trend toward a reduced risk of major adverse cardiovascular events. On the other hand, a recent meta-analysis of our groups [48] stated the superiority of rate control strategy, in patient with non rheumatic atrial fibrillation, in reducing the risk of the combined end point of all cause death and thromboembolic stroke without any increase in the risk of major haemorrhages. This superiority seems even more evident by the low number needed to treat to avoid one combined end point as it results equal to 50. Rate control strategy confirmed its superiority in reducing the combined end point compared to the rhythm control approach  even when older patient or longer follow up were considered. Notably, the risk of thromboembolic stroke is strongly reduced  in the early period after the beginning of therapy as demonstrated in the studies with mean follow up < 20 months in which we observed a reduction of 82%.

## Conclusions

In the evidence-based medicine era the highest level of evidence is that of meta-analytic approach [49] but the results presented above should be cautiously viewed as hypothesis generating not as the definitive answer, for some reasons: 1) the results do not apply to all subsets of patients with atrial fibrillation because patients with Wolff-Parkinson-White syndrome, those who had previously undergone heart surgery, or those with NYHA class IV heart failure were excluded by the trials’ designs; 2) all published studies had at least a small percentage of patients crossing over the randomization arms but the statistical analyses were performed by intention to treat thus underestimating this effect, and 3) even if amiodarone was the antiarrhythmic drug more frequently used, other agents have been adopted, anyone with its own risk/benefit profile, thus  generating another confounding factor to take into account.

In a post hoc analysis of AFFIRM population the trend toward a lower total mortality in the rate-control versus the rhythm-control group appears entirely explained by non cardiovascular deaths. Independent predictors of non cardiovascular death were rhythm-control strategy, age, male gender, previous smoking history, heart failure, and coronary heart disease [50]. In another analysis, the authors stated that digoxin and antiarrhythmic drugs were directly associated, while the presence of sinus rhythm and warfarin therapy were inversely associated with increased mortality after adjustment for other covariates [51]. The meaning of these separate observations may be that pursuing sinus rhythm may improve prognosis, but this potential advantage has to be weighted against various non cardiovascular adverse effects of the agents used to obtain and maintain it.

As previously stated, current guidelines recommend only a short term anticoagulation therapy after restoration of sinus rhythm while those patients treated with rate control approach continue to take Vitamin K antagonists or aspirin potentially for all the life. This issue rises some concerns: oral anticoagulants, but also aspirin, are associated with at least a two fold risk of major haemorrhages compared to placebo [13], on the other hand, current available antiarrhythmic drug are burdened by a substantial risk of ventricular arrhythmias. A patients treated with rhythm control approach have not an increased risk of haemorragies but an increased risk of sudden death. The contrary may be told for those treated with rate control strategy. In these patients with atrial fibrillation, with so many different clinical features, what is the highest and worst risk? An haemorrhagic stroke or a malignant arrhythmia? We have not enough evidence to answer this pivotal question.

As previously reported [48], there is an early large excess of thromboembolic stroke in patients randomized to a rhythm-control strategy possibly because the risk of stroke associated with electrical cardioversion is not entirely abolished by short term anticoagulation. Moreover, symptomatic or asymptomatic recurrences are frequent [1] thus increasing the risk of thromboembolic stroke in patients possibly taking neither aspirin nor Vitamin K antagonists.

In the future, new, safer and more effective antiarrhythmic agents, associated with careful and prolonged anticoagulation, will probably make the rhythm-control strategy superior to the rate control one but, according to current evidence, rate-control strategy represents the gold standard strategy, especially for those patients with echocardiographic and clinical features that make unlikely the maintenance of sinus rhythm with or without antiarrhythmic prophylaxis for recurrences.
